# One‐Step Immobilized Phage Depolymerase Coatings for Catheter‐Related Bloodstream Infections

**DOI:** 10.1002/advs.77319

**Published:** 2026-08-21

**Authors:** Lingkai Dong, Xingjin Li, Tao Hu, Qianqian Lu, Xiaoxu Zhang, Xi Chen, Yifan Luo, Zhong Zuo, Ronald Man Yeung Wong, Miao Xu, Tiancong Zhao, Sharon Shui Yee Leung

**Affiliations:** ^1^ School of Pharmacy The Chinese University of Hong Kong Shatin Hong Kong China; ^2^ College of Smart Materials and Future Energy Department of Chemistry Fudan University Shanghai China; ^3^ Institutes of Biomedical Sciences Shanghai Key Laboratory of Medical Epigenetics Fudan University Shanghai China; ^4^ Department of Chemistry The Chinese University of Hong Kong, Shatin Hong Kong China; ^5^ Department of Orthopaedics & Traumatology The Chinese University of Hong Kong Hong Kong China

**Keywords:** antibacterial coatings, CRBSI, multidrug‐resistant bacteria, phage‐derived depolymerase

## Abstract

Catheter‐related bloodstream infection (CRBSI) is an important clinical problem that causes significant mortality and excess economic cost. Recent epidemiological trends indicate a shift in CRBSI pathogens from Gram‐positive to Gram‐negative bacilli, among which *Pseudomonas aeruginosa*, *Klebsiella pneumoniae*, and *Acinetobacter baumannii* predominate due to their strong antibiotic resistance and biofilm‐forming capabilities. Recently, bacteriophage‐encoded depolymerases have emerged as promising antivirulence agents that resensitize bacteria to host immunity by degrading capsular polysaccharides (CPS) and exopolysaccharides (EPS). In this study, we employed a mussel‐inspired polydopamine (PDA) coating technique to immobilize an *A. baumannii‐specific* depolymerase, DPO71, onto diverse organic, inorganic, and metal substrates, with a maximum surface coverage of​ ∼0.2 µg/cm^2^. In the presence of human serum, DPO71‐coated surfaces demonstrated potent antibacterial and antibiofilm activities. The coating significantly reduced bacterial burden in an epithelial cell‐bacteria co‐culture system and in ex vivo blood infection models. Moreover, DPO71 coatings demonstrated good robustness, stability, and minimal toxicity toward epithelial cells and *Galleria mellonella* larvae​. Importantly, in a mouse subcutaneous implantation model, the DPO71 coating significantly reduced bacterial load in vivo. Overall, modifying catheter surfaces with depolymerases via a simple PDA coating technique demonstrates great potential in mitigating bacteria‐associated CRBSI.

## Graphical abstract

Catheter‐related bloodstream infections (CRBSI) caused by drug‐resistant Gram‐negative bacteria remain a major clinical challenge. Here, a polydopamine‐assisted depolymerase coating degrades bacterial capsules and restores serum‐mediated killing, effectively suppressing biofilms and reducing bacterial burden in vitro, ex vivo, and in vivo, presenting a promising approach to manage CRBSI. (The graph is created with Biorender.com)

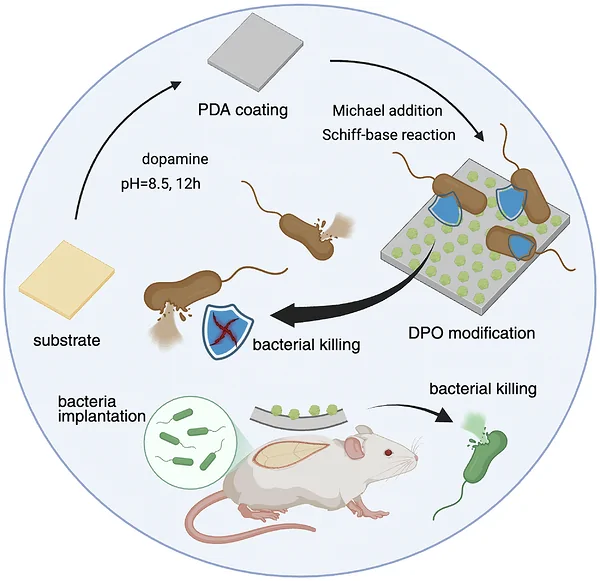

## 1 Introduction

Catheter‐related bloodstream infection (CRBSI) is the most common cause of nosocomial bacteremia [1, 2]. *Staphylococcus aureus* was the major causative pathogen for CRBSI in the past, but recent epidemiology data showed a linear shift toward Gram‐negative (G‒ve) bacteria [3, 4], particularly *Pseudomonas aeruginosa*, *Klebsiella pneumoniae*, and *Acinetobacter baumannii*, which are included in the two Bacterial Priority Pathogens Lists published by the World Health Organization [5]. With such a shift, the 30‐day mortality rate of CRBSI has elevated to ≥ 25% [4]. Common features of these priority pathogens are their excellent capabilities in forming biofilms and acquiring resistance against antibiotics, rendering most current antibiotic treatments ineffective. In this context, there is an urgent need to develop new strategies to prevent CRBSI [6, 7, 8, 9].

Surface modification is an effective strategy to mitigate CRBSI [10, 11, 12]. Antimicrobial coatings are divided into three types according to their strategies to eradicate bacteria: (i) anti‐adhesive coatings preventing bacteria adhesion and biofilm formation; (ii) bactericide‐releasing coatings; and (iii) contact‐killing coatings where bactericidal agents are conjugated to the surface [13, 14]. While the anti‐adhesive coatings rely on steric, electrostatic repulsion, or a hierarchical topography to repel bacteria rather than killing them, they might have a limited effect against bacteria with good adhering capabilities, and the performance might reduce with time easily [15]. As for the bactericide‐releasing coatings, it is difficult to maintain the released antibacterial agents within the effective range throughout the application period, leading to the concern of promoting resistance development due to the suboptimal levels of antibacterial agents [16]. On the other hand, the contact‐killing coatings do not release biocides into body fluids. Instead, the immobilized antibacterial agents (like quaternary ammonium compounds, peptides, and phages) kill bacteria adhered to the surface or those from the surroundings, minimizing the chance of resistance development [17, 18, 19]. Although quaternary ammonium compounds [17] and antimicrobial peptides [18] have been widely investigated for use as contact‐killing coatings, their positively charged nature is likely to promote protein adsorption, reducing the antibacterial efficacy. In addition, the leachable contents may impose toxicity issues [20, 21, 22].

Phage‐encoded depolymerases have recently been considered as promising antimicrobial agents as they are biocompatible and can effectively degrade bacterial polysaccharides, including the capsular polysaccharide (CPS), lipopolysaccharide (LPS) and extracellular polysaccharide (EPS), which confer the virulence of bacteria and the biofilm formation capability [23, 24, 25]. To date, coating surfaces with depolymerases has not been attempted, possibly because depolymerases do not directly kill bacteria but merely disintegrate the CPS to expose bacteria for host immune attack [26, 27]. Effective bacteria killing in the presence of serum/ serum compliment has been demonstrated in recent studies [25, 28, 29]. The rich serum content in blood suggests that depolymerase‐coated catheters may prevent CRBSI. Despite that modification of catheter surfaces with proteins could serve as an effective strategy because protein adsorption is the first step when biomaterials and medical devices are implanted in the human body [30], immobilizing proteins onto material surfaces can be challenging due to potential deactivation of proteins upon their adsorption onto the surfaces, particularly under harsh conditions such as high temperature and extreme pH [31, 32, 33].

**FIGURE 1:**
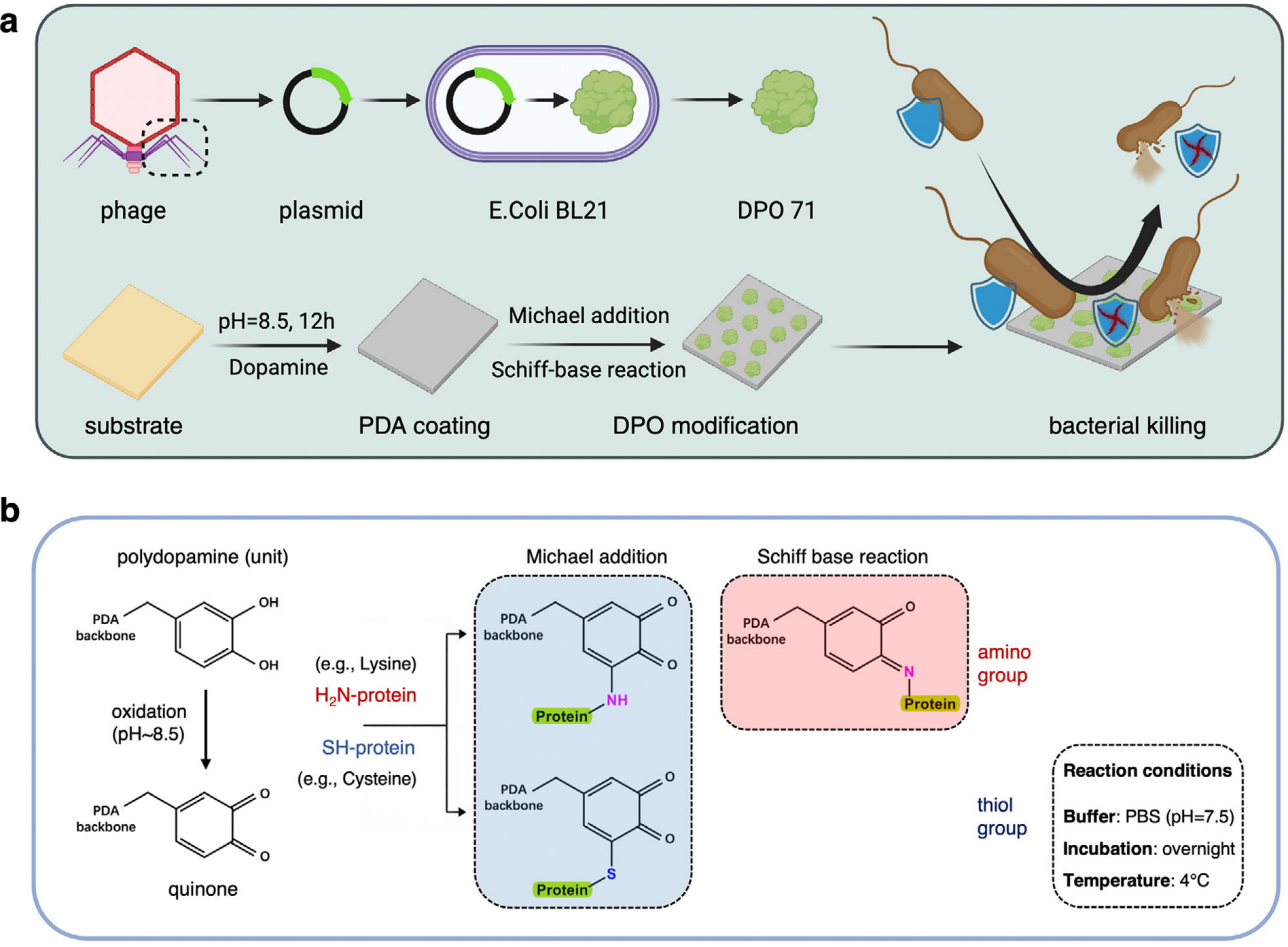
Schematic illustration of the fabrication procedure of DPO‐based coatings that endow the surface with antibacterial activity. Created with Biorender.com. (a) Recombinant protein obtained using an *E. coli* expression system and PDA coating procedures for DPO immobilization. (b) Schematic diagram of the Schiff base reaction and Michael addition reaction between the PDA coating and proteins (e.g., DPO71).

Recently, mussel‐inspired polydopamine (PDA) chemistry has opened a new avenue for surface modification, particularly for biological species, due to its mild reaction conditions, simplicity, and high effectiveness [34, 35, 36]. In the present study, a depolymerase derived from an *A. baumannii* phage (vB_AbaM‐IME‐AB2), namely DPO71 with excellent stability [37], was used as the model depolymerase. The feasibility of immobilizing purified recombinant DPO71 onto various surface materials via the PDA approach was first examined using a protocol sketched in Figure 1. The Schiff base formed between the quinone carbonyl group of the PDA coating and the ε‐primary amine group of a protein lysine residue is reversible in an aqueous environment. In contrast, covalent adducts formed through Michael addition reactions between nucleophilic groups such as –NH2 or –SH in proteins and the PDA backbone are essentially irreversible under physiological conditions. Therefore, under the combined effects of Michael addition and Schiff base reactions enable stable multi‐point covalent anchoring of protein molecules on PDA‐coated surfaces. Following overnight incubation, this immobilization process results in robust attachment that is nearly irreversible under physiological conditions.

The antibacterial and antibiofilm properties and the biocompatibility of DPO71‐coated surfaces (DPO coatings) against an MDR strain of *A. baumannii*, MDR‐AB2, were then evaluated. The applicability of the DPO71 coatings in mitigating CRBSI was further verified in a bacterial‐epithelial cell co‐culture system, ex vivo blood infection, and circulation models. Finally, the in vivo applicability of the DPO71 coating against implant infection was evaluated using a mouse subcutaneous implantation model.

## 2 Materials and Methods

### 2.1 Materials

Sodium hydroxide (NaOH), phosphate‐buffered saline (PBS), ethanol, bovine serum albumin (BSA), Sulfo‐Cy5, and 3‐Hydroxytyramine hydrochloride (dopamine hydrochloride) were purchased from Aladdin (Shanghai, China). Anti‐IgG HRP was purchased from Biosharp (China). The SYTO 9 fluorescent nucleic acid dye, LIVE/DEAD Bacterial Staining Kit with DMAO & PI, and LIVE/DEAD Staining Kit with Calcein‐AM/PI for cell staining were purchased from Beyotime Biotechnology (Shanghai, China). Luria‐Bertani (LB) agar and Nutrient Broth (NB) were obtained from Sigma–Aldrich (St. Louis, USA). Human serum (H4522) used in this study was purchased from Sigma–Aldrich (Saint Louis, Missouri, United States). An MDR strain of *A. baumannii* (MDR‐AB2) isolated from a pneumonia patient at PLA Hospital 307 was gifted from Beijing Institute of Microbiology and Epidemiology.

### 2.2 Depolymerase Expression and Purification

Depolymerase DPO71 (Protein ID: YP_009592222.1) encoded from an *A. baumannii* phage was employed as the model depolymerase. The constructed pET28a plasmid, consisting of genes encoded DPO71 and an anti‐kanamycin gene, was transformed into *E. coli* BL21 (DE3) cells. The transformed *E. coli* was grown overnight at 37°C in LB medium supplemented with 50 µg/mL kanamycin. The bacterial cells were then lysed in lysis buffer (10 mm imidazole, 50 mm phosphate/300 mm sodium chloride) by sonication and followed by centrifugation. The collected supernatant was filtered and loaded into a HisTrap HP column (GE Healthcare, Chicago, USA) and eluted with an imidazole gradient from 10 mm to 500 mm. Purified protein was flash‐frozen using liquid nitrogen and stored at ‒80°C. The concentration of the protein was measured using the BCA Protein Assay Kit (Thermo Fisher Scientific, Massachusetts, USA).

### 2.3 Preparation of PDA and DPO Coatings

The deposition of PDA and immobilization with DPO71 were prepared as described previously for protein coating with minor modification [38]. Briefly, dopamine (2 mg/mL) was dissolved in Tris‐HCl solution (pH = 8.5, 10 mm). Multiple materials, titanium (Ti) plate, polyurethane (PU) plate or tube, glass disc (SiO_2_), and 24‐well cell culture plate, were cleaned with anhydrous ethanol and dried. Next, various materials were immersed in the dopamine solution overnight at 37°C, followed by sonication and washing with PBS three times to remove residual reactants. Subsequently, all PDA coated substrates, except the 24‐well plates, were incubated with three different concentrations of DPO71 solutions (DPO‐A: 0.01 mg/mL; DPO‐B: 0.02 mg/mL; and DPO‐C: 0.1 mg/mL) in PBS at 4°C for 12 h for surface immobilization. For coating DPO71 onto 24‐well cell culture plates, each well was incubated with 0.2 mL of protein solutions at varying concentrations, corresponding to theoretical surface coverage densities of 4 µg/cm^2^ (DPO4), 2 µg/cm^2^ (DPO2), 1 µg/cm^2^ (DPO1), and lower as indicated. The modified samples were then rinsed with PBS thrice before characterization. BSA, Sulfo‐Cy5‐labelled BSA, and anti‐IgG HRP, instead of DPO71, were used in some experiments in characterizing the coating efficiency and robustness (specified in individual tests). The protein loading was roughly assessed by the difference in fluorescence intensity in the supernatant before and after the reaction, and precise quantification of protein coatings was achieved by measuring the changes in OD values ​​after HRP catalysis of the substrate. The prepared samples were named according to the substrate and coating, such as SiO_2_, PDA@SiO_2_, and BSA@PDA@SiO_2_.

### 2.4 Physicochemical Characterization of DPO Coatings

The morphologies of bare and coated surfaces were characterized by scanning electron microscopy (SEM) and atomic force microscopy (AFM, Seiko, SPI‐380N, Japen). Chemical composition and molecular structure were analyzed using x‐ray photoelectron spectroscopy (XPS, Thermo Fisher Scientific, USA) and Raman scattering spectroscopy, respectively. Water contact angle (WCA) was detected using a DropMeter A‐200 contact angle system (MAIST Vision Inspection & Measurement Co. Ltd., China). The surface coverage of Sulfo‐Cy5‐labeled BSA on various surfaces was examined using a fluorescence microscope (NIKON, Japan).

### 2.5 Contact Killing Assay of the DPO Coated Samples With Human Serum

Mid‐log phase bacteria were resuspended in a mixture of PBS and serum with a serum volume ratio of 0%−20%. A volume of 200 µL of the bacteria suspension was pipetted and spread onto the uncoated (control), PDA‐coated, and DPO‐coated 24‐well plates. After incubating at 37°C for 2 h, residual bacteria were recovered with PBS by thorough pipetting, followed by 10‐fold dilution for colony formation unit (CFU) counting. The kinetics of the contact killing assay was also performed with 20% serum. All experiments were performed in triplicate.

The morphological changes of the bacteria on these surfaces were examined using field emission scanning electron microscopes (FESEM, QUANTA 200 PLUS, Christ, Germany). Briefly, after incubation, all samples were washed with PBS three times to remove any loosely attached bacteria. The adhered bacteria were then fixed with 4% paraformaldehyde for 1 h and dehydrated with gradient ethanol solutions. The samples were coated with gold before FESEM observation.

### 2.6 Biofilm Inhibition Activity

A volume of 400 µL mid‐log phase *A. baumannii* culture (OD_600_ = 0.13) was pipetted onto the uncoated (control), PDA‐coated, and DPO‐coated surfaces in a 24 well‐plate and incubated at 37°C for 2 and 4 h. Then, the bacteria suspension was removed, and 0.5 mL NB medium with 20% serum was added to each well for further incubation. After 4 h, the planktonic bacteria were removed. Then, the surfaces were rinsed thrice with PBS. Next, biofilms were treated with Crystal Violet (CV) staining to determine the biofilm biomass. Treated biofilms were also stained with SYTO 9 green fluorescent nucleic acid dye and LIVE/DEAD Bacterial Staining Kit with DMAO & PI for confocal laser scanning microscopy and fluorescent microscopy observation.

### 2.7 Stability of DPO Coating Against Ultrasonication

Protein‐coated 24‐well plates were added with 1 mL PBS and sonicated for 0, 1, or 2 min at 50 Hz, 200 W. Then, PBS was removed, and their antimicrobial activity was evaluated in the presence of 20% serum. The surface coverage of Cy5‐BSA on the surfaces before and after sonication was observed under fluorescence microscopy to evaluate the coating stability against mechanical stress. The HRP‐coated 24‐well plates were used for protein quantification before and after sonication via ELISA. The antibacterial activity of sonicated DPO‐coated plates was also determined by plate counting.

### 2.8 Nano‐Scratching Assay

BSA@PDA@PU was prepared as described above and used for nano‐scratching testing. Nano‐scratching experiments were performed using a TriboIndenter (TI 950, Hysitron, USA) equipped with a Berkovich diamond tip. Briefly, the scratching was conducted under a linearly increasing normal load from 0 to 200 µN over a scratch length of 10 µm at a constant speed. Normal and lateral forces with time were recorded to generate force‐displacement profiles. The resulting scratch tracks were subsequently imaged by optical microscopy to evaluate coating adhesion and deformation behavior.

### 2.9 Hemolysis and Cytotoxicity of DPO Coating

Rat whole blood was supplied by the Laboratory Animal Research Center (The Chinese University of Hong Kong). A volume of 1 mL of fresh rat red blood cells (RBCs) was centrifuged (4000 rpm, 10 min) and the supernatant was discarded. The RBCs pellet was then washed three times and diluted to a concentration of 5% (v/v) with PBS. A volume of 1 mL of the diluted RBCs was added to the uncoated, PDA‐coated, and DPO‐coated 24‐well plates. PBS and 0.1% Triton X‐100 were served as the negative and positive controls, respectively. All samples were incubated at 37°C with 150 rpm shaking for 2 h. After incubation, all samples were centrifuged for 10 min, and 100 µL of the supernatant was pipetted into a 96‐well plate. The absorbance at 545 nm was measured with a microplate reader. The percentage of hemolysis was calculated according to the equation below:

$$\%\;hemolysis=\frac{A\left(\mathrm{sample}\right)-A\;\left(\mathrm{negative}\right)}{A\left(\mathrm{positive}\right)-A\left(\mathrm{negative}\right)}\;\;\times 100\%$$

Human lung cancer epithelial cells (A549) were cultured in Roswell Park Memorial Institute 1640 (RPMI 1640) supplemented with 10% (v/v) fetal bovine serum (FBS) in a humidified atmosphere containing 5% CO_2_ at 37°C. A549 cells were seeded in 24‐well plates that were modified with PDA and different concentrations of DPO71. The cellular metabolic activity on the coatings was measured by the CCK‐8 assay after 1 and 2 days. The morphologies of epithelial cells on the coatings were determined by Calcein‐AM/PI dye.

### 2.10 Antimicrobial Assay of the DPO Coatings in an Epithelial Cell and Bacteria Co‐Culture System

A volume of 20 µL of *A. baumannii* suspension (10^8^ CFU/mL) was added to wells modified with PDA and DPO coatings. The uncoated wells were served as negative controls. After 2 h of incubation with the bacteria, A549 cells in 1 mL RPMI 1640 with 10% FBS were added to each well and incubated in a humidified atmosphere with 5% CO_2_ at 37°C for overnight. Following co‐incubation, the culture medium was first collected for optical density (OD) measurement to assess bacterial growth in the supernatant. The morphologies of cells on different surfaces were then observed under a fluorescent microscope. Subsequently, each well was gently rinsed three times with PBS to remove non‐adherent bacteria. To quantify the adherent and intracellular bacteria, the cells were lysed with 0.1 % Triton X‐100 in PBS for 5 min at 37°C with gentle shaking. The resulting lysates were serially diluted (tenfold), plated onto LB agar plates, and incubated overnight at 37°C for CFU counting.

### 2.11 Antimicrobial Efficiency of the DPO Coating in an Ex Vivo Blood Infection Model

The samples (uncoated, PDA‐coated, and DPO3‐coated surfaces) were added 20 µL *A. baumannii* suspension (10^8^ CFU/mL), and all samples were added 0.5 mL rat whole blood with shaking (200 rpm) for 2 h incubation in a humidified chamber at 37°C. CFU counting in the culture medium was performed as described above. One sample from different group was fixed with 4% formaldehyde for 4 h and then dehydrated with ethanol gradient solutions and sputtered with 15 nm gold for morphology examination using FESEM.

An ex vivo circulation system was established to simulate blood flow conditions. Briefly, 4 mL medium containing 20% serum or 4 mL rat whole blood was circulated continuously through uncoated or DPO‐coated catheters. The flow was driven by a peristaltic pump (Gilson, Minipuls 3, Spain) at a constant rate comparable to that used during clinical infusion. At the start of circulation, 1 × 10^6^ CFU of bacteria were introduced into the system. After 24 h of continuous circulation, the liquid was collected for CFU counting.

### 2.12 Animal Models

*Galleria mellonella* larvae were used to evaluate both the in vivo safety of DPO71 and its therapeutic efficacy against bacterial infection. To assess in vivo safety, 20 µL of PBS containing either 4 µg DPO71 or 20% serum (4 µL serum diluted in PBS) was injected. For infection treatment studies, DPO71 was dissolved in PBS containing different concentrations of serum, mixed with a bacterial suspension (1 × 10^6^ CFU), and immediately injected into the larvae.

In addition, the in vivo applicability of the DPO71 coating for preventing implant‐associated infection was evaluated using a mouse subcutaneous implantation model using BALB/c mice (18–22 g). All animal procedures were approved by the Animal Experimentation Ethics Committee (AEEC) of CUHK (Ref No.: 22–124–HMF). Briefly, catheter fragments pre‐incubated with bacteria (1 × 10^7^ CFU) were implanted subcutaneously into the dorsal region of mice. Body weight was recorded every two days as an indicator of systemic health. On day 10 post‐implantation, mice were euthanized and surrounding skin tissues were harvested. A portion of the tissues was fixed in 4% paraformaldehyde, embedded in paraffin, and sectioned for H&E staining and Masson's trichrome staining. The remaining tissue samples were processed for cytokine expression analysis and bacterial burden quantification using RT‐qPCR.

### 2.13 RNA Extraction, Reverse Transcription, and RT‐qPCR

Total RNA was extracted from harvested tissue samples using TRIzol reagent according to the manufacturer's instructions. RNA concentration and purity were measured using a NanoDrop 2000 spectrophotometer (Thermo Fisher Scientific). cDNA was synthesized up to 1 µg of total RNA using the PrimeScript RT reagent Kit with gDNA Eraser (Takara). RT‐qPCR was performed in 384‐well plates using TB Green Premix Ex Taq (Tli RNaseH Plus) on a LightCycler 480 Instrument II (Roche). Each 10 µL reaction mixture contained 5 µL TB Green Premix, 1 µL diluted cDNA template, 2 µL primer mix (Table S1), and 2 µL ddH_2_O. Amplification was conducted with an initial denaturation at 95°C for 30 s, followed by 40 cycles of denaturation at 95°C for 10 s and annealing at 60°C for 30 s. Relative gene expression levels were calculated using the 2‐ΔΔCt method with GAPDH serving as the internal gene.

### 2.14 Statistical Analysis

All data were presented as mean ± standard deviation unless otherwise specified. GraphPad Prism was used to conduct statistical analysis. Unpaired Student's t‐test was used to compare two groups, and one‐way analysis of variance (ANOVA) was used to compare multiple groups. P values lower than 0.05 (^*^ *p* < 0.05) were considered the threshold for statistical significance among indicated groups.

## 3 Results and Discussion

### 3.1 Characterization of the PDA and Protein Coating

Polydopamine (PDA) deposition and subsequent protein modification, with BSA as a model protein, were performed on various materials, including polyurethane (PU), titanium (Ti), and glass (SiO_2_), to evaluate the universality of the PDA coating strategy. A uniform black film was observed on all material surfaces after PDA deposition, indicating successful formation of a PDA layer (Figure S1). This characteristic dark coloration originates from the oxidative self‐polymerization of dopamine under mildly alkaline conditions, which generates melanin‐like PDA structures composed of conjugated polycatecholamine and indole units with strong broadband light absorption [39]. The spontaneous formation of this black PDA film across chemically distinct substrates reflects the universal adhesion capability of PDA, arising from the synergistic interactions of catechol and amine groups with organic polymers, inorganic oxides, and metallic surfaces through hydrogen bonding, *π–π* stacking, cation–π interactions, and metal coordination [40]. Following PDA deposition, protein molecules were immobilized onto the PDA‐coated surfaces primarily through Michael addition and Schiff base reactions between nucleophilic amine or thiol groups in proteins and the quinone moieties present in the PDA layer [41]. Different from the PDA coating inducing distinct surface color changes, subsequent protein immobilization caused no additional macroscopic visual differences (Figure S2).

**FIGURE 2:**
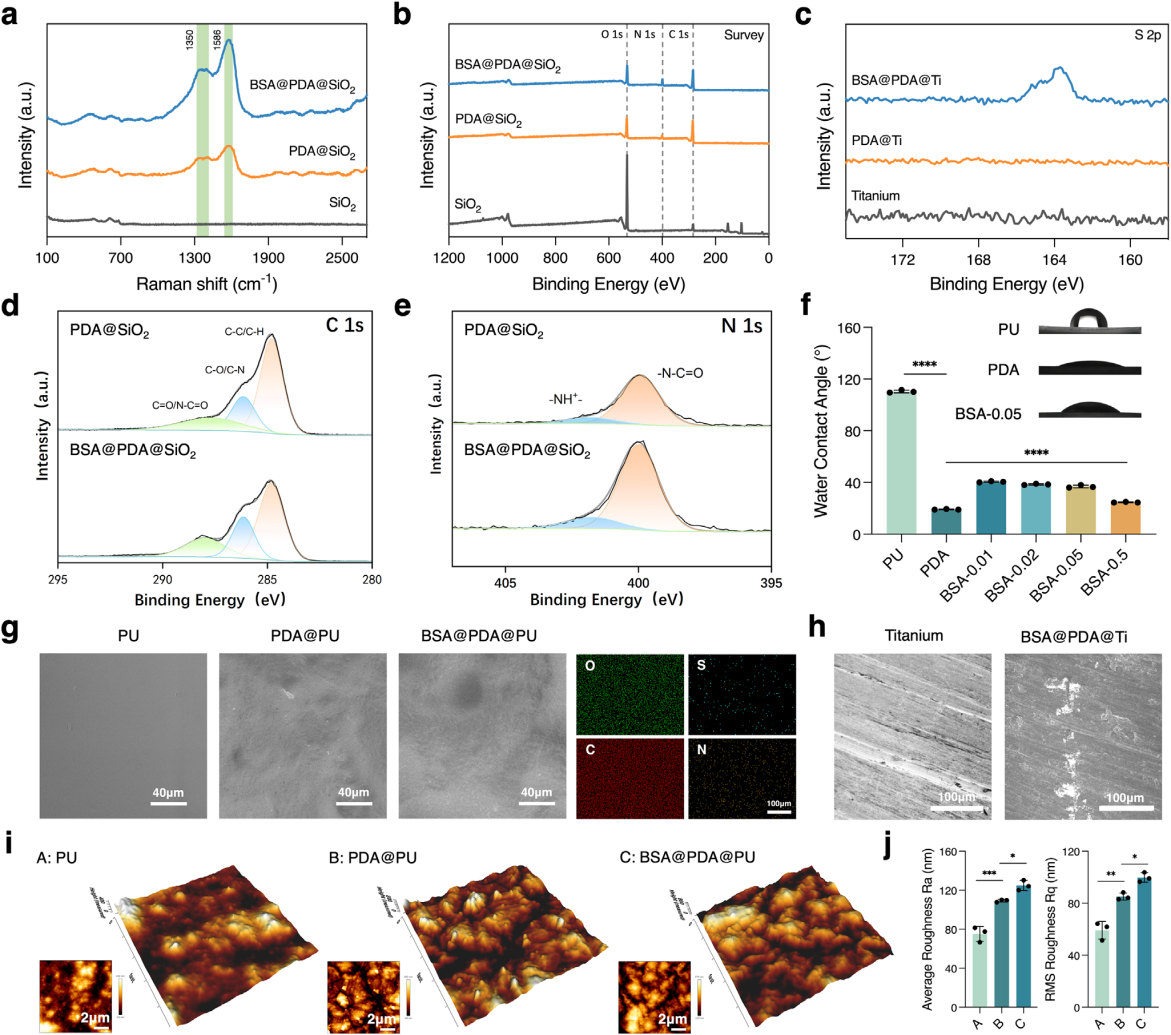
Characterization of the PDA and protein coatings. (a) Raman scattering results of glass (SiO_2_), PDA@SiO_2,_ and BSA@PDA@SiO_2_. (b) Survey of the XPS signals from SiO_2_, PDA@SiO_2_, and BSA@PDA@SiO_2_. (c) The XPS results of S 2p from titanium plate (Ti), PDA@Ti and BSA@PDA@Ti. (d) High‐resolution XPS spectra of C 1s from PDA@ SiO_2_ and BSA@PDA@ SiO_2_. (e) High‐resolution XPS spectra of N 1s from PDA@ SiO_2_ and BSA@PDA@ SiO_2_. (f) Water contact angle (WCA) measurement of PU plate before and after PDA and protein coating. (g) SEM imaging (Scale bar = 40 µm) of the surface morphology from SiO_2_, PDA@SiO_2_, and BSA@PDA@SiO_2_, and EDS mapping (Scale bar = 100 µm) of the BSA@PDA@SiO_2_ surface. (h) SEM imaging (Scale bar = 100 µm) of the surface morphology from Ti and BSA@PDA@Ti. (i) AFM images (Scale bar = 2 µm) of surfaces of the PU, PDA@PU, and BSA@PDA@PU. (j) Average roughness Ra and RMS roughness Rq of the surfaces of PU, PDA@PU, and BSA@PDA@PU. Data are presented as mean ± s.d. (*n* = 3 independent samples) and analyzed with one‐way ANOVA followed by Tukey's multiple comparisons test. ^*^p < 0.05, ^**^p < 0.01, ^***^p < 0.001, and ^****^p < 0.0001.

The coating performance on various material surfaces was characterized at the molecular level. Raman spectroscopy revealed that after PDA deposition on the glass surface, two prominent peaks appeared at Raman shifts of ∼1350 cm^−^^1^ and ∼1586 cm^−^^1^, which correspond to the D and G bands of PDA arising from disordered carbon structures and sp^2^‐hybridized aromatic domains, respectively, confirming successful PDA coating (Figure 2a). These peaks were further enhanced after protein immobilization, likely due to increased local polarizability and Raman scattering at the PDA–protein interface rather than the formation of new vibrational modes.

X‐ray photoelectron spectroscopy (XPS) was also employed to evaluate PDA and protein coatings (Figure 2b–e). On the glass surface, PDA and DPO coatings resulted in a decreased O 1s signal and a pronounced increase in the N 1s signal, consistent with the nitrogen‐rich PDA and protein layers (Figure 2b). As for the titanium substrate, PDA deposition effectively attenuated the Ti 2p signal to an undetectable level, while the appearance of an S 2p signal after protein modification was attributed to disulfide bonds present in BSA (Figure 2c and Figure S3). In contrast, PDA and BSA coatings on a polyurethane (PU) plate did not induce significant changes in the XPS survey spectra (Figure S4), possibly because of the similar elemental compositions of the PU substrate, PDA, and protein. To further verify protein immobilization on the glass surface, high‐resolution XPS spectra were analyzed. After protein modification, enhanced C═O/N−C═O signals in the C 1s spectra and an increased −NH^+^ signal in the N 1s spectra were observed, confirming the formation of a protein coating on the PDA layer (Figure 2d,e). Similar spectral changes were also observed for PDA‐coated titanium and PU substrates following protein modification (Figures S5 and S6), indicating the general applicability of this surface functionalization strategy.

Hydrophobicity of PU surfaces coated with different amounts of protein was evaluated by measuring water contact angle (WCA). As shown in Figure 2f, PDA coating dramatically reduced the WCA of the PU from ∼110° to ∼19°, owing to the abundant hydrophilic amino and hydroxyl groups present in the PDA layer. Subsequent immobilization of BSA increased the WCA of the coated surface at a low BSA concentration (0.01 mg/mL), while higher protein concentrations led to a gradual decrease in WCA, indicating concentration‐dependent surface coverage by the protein layer. SEM imaging revealed that the surface morphology became rougher with enhanced contrast after PDA and DPO coating on PU and glass plates, attributing to the improved surface conductivity provided by the PDA layer (Figure 2g and Figure S7). Elemental mapping by energy‐dispersive x‐ray analysis (EDS) confirmed the uniform distribution of protein on the BSA@PDA@PU surface (Figure 2g). For titanium substrates, the bare surface displayed clear metallic textures, whereas continuous film coverage was observed after PDA and protein coating (Figure 2h). Atomic force microscopy (AFM) further revealed increased surface roughness after PDA deposition and protein immobilization on the PU substrate, which was attributed to localized, nonuniform PDA deposition and subsequent protein coverage (Figure 2i). Quantitative analysis confirmed a significant increase in surface roughness after each modification step (Figure 2j). In addition, fluorescence microscopy of sulfo‐Cy5–labeled BSA directly visualized successful protein immobilization, with the fluorescence distribution closely following the underlying PDA coating morphology (Figure S8). Collectively, these results confirmed that the mussel‐inspired PDA functionalization strategy can effectively immobilize protein molecules onto diverse material surfaces.

Building on this validation of the PDA‐based coating strategy, BSA was subsequently replaced with DPO71 to evaluate the functional properties of the depolymerase coating (Figure S9). Consistent with the BSA model system, both PDA and DPO coatings exhibited strong Raman signals (Figure S9a). XPS analysis also revealed changes in elemental composition following DPO immobilization, with high‐resolution C 1s spectroscopy showing distinct alterations in carbon composition, confirming successful DPO deposition (Figure S9b–d). SEM imaging provided a clear visualization of the PDA and DPO coatings, while EDS mapping demonstrated the uniform elemental distribution of the DPO layer (Figure S9e,f).

### 3.2 Antibacterial Activity of the DPO Coatings

**FIGURE 3:**
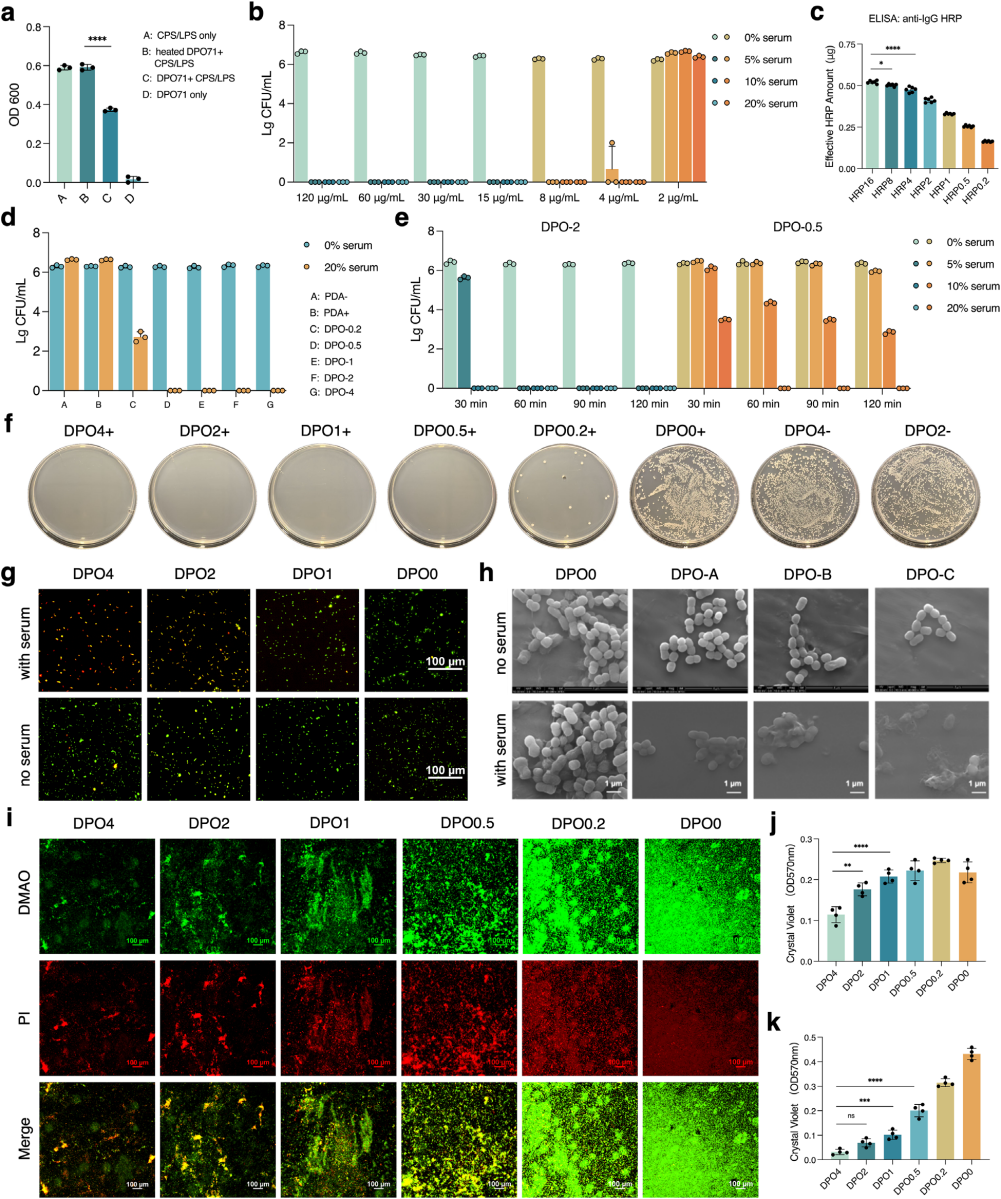
Antibacterial activity of DPO coatings. (a) The ability of depolymerases degrading CPS/LPS extracts. (b) Antibacterial activity of DPO solution with different concentrations in the presence of different serum concentrations (0%, 5%, 10%, and 20%). (c) Quantification of protein coating via ELISA. The number after HRP denoted the incubation protein concentration used in the coating reaction. (Figure 3c). (d) The antibacterial activity of DPO coating in a 24‐well plate with or without 20% serum. (e) Antibacterial activity of DPO coating in the presence of different serum concentrations (0%, 5%, 10%, and 20%) at different time points. (f) Photos of bacterial colonies from different groups; DPO+ means 20% serum, and DPO‐ means no serum. (g) Antibacterial activity of DPO coatings in the presence of 10^8^ CFU of bacteria. (Scale bar = 100 µm). (h) Morphologies of bacteria on the PDA or DPO‐coated surfaces in the absence or presence of serum. (Scale bar = 1 µm.) (DPO0: PDA only; DPO‐A: incubation with 0.01 mg/mL DPO solution; DPO‐B: 0.02 mg/mL and DPO‐C: 0.1 mg/mL) (i) Anti‐biofilm activity of the PDA or DPO coatings with 20% serum. (Scale bar = 100 µm.) (j) Quantification of CV‐staining biofilm at 2 h after the bacteria were added to the DPO coating. (k) Quantification of CV‐staining biofilm at 4 h after the 20% serum was added to the DPO coating after biofilm formation. Data are presented as mean ± s.d. (*n* = 3, 4, or 6 independent samples) and analyzed with one‐way ANOVA followed by Tukey's multiple comparisons test. ^*^p < 0.05, ^**^p < 0.01, ^***^p < 0.001, and ^****^p < 0.0001.

The bioactivity of purified recombinant DPO71 was first evaluated (Figure 3a). The results confirmed that DPO71 effectively degraded CPS/LPS extracted from the host bacteria of the parent phage, while heat‐inactivated DPO71 (90°C for 15 min) completely lost its polysaccharide‐degrading activity. Although depolymerases do not directly kill bacteria, they could effectively sensitive serum killing even at low serum levels down to 5% [37]. Before evaluating the antibacterial performance of the DPO coating, we therefore examined the bactericidal ability of DPO71 solutions at different concentrations under serum concentrations ranging from 0% to 20%. The results showed that when the DPO concentration exceeded 4 µg/mL, the presence of 5% serum was sufficient to completely kill bacteria within 2 h. In contrast, when the DPO71 concentration was reduced to 2 µg/mL, even 20% serum failed to achieve effective bacterial killing (Figure 3b). These findings indicate that the antibacterial ability is jointly dependent on both DPO and serum concentrations.

Subsequently, DPO71 was immobilized onto different surfaces using the mussel‐inspired PDA chemistry. Two approaches were employed to quantify the protein loading on the coated surfaces. First, by measuring the change in protein concentration in solution before and after the reaction. The loading was estimated to be less than 0.5 µg/cm^2^ (Figure S10). However, this method may overestimate the actual loading due to nonspecific protein adsorption. To obtain a more accurate measurement, anti‐IgG HRP was used as a model protein, and the surface loading was quantified using an ELISA. Results showed that protein loading increased with increasing incubation protein concentration but approached a saturation plateau at approximately 0.2 µg/cm^2^, indicating a nonlinear adsorption behavior rather than a linear increase (Figure 3c). The observed saturation of surface‐immobilized protein suggests that the PDA‐modified surface provides a finite number of binding sites, aligning with surface‐limited immobilization mechanisms.

To function as an effective antibacterial coating, it is essential that the immobilized DPO71 retains its bioactivity. Accordingly, we evaluated the antimicrobial performance of coatings containing different amounts of DPO71 (DPO0.2−DPO4) using DPO‐coated 24‐well plates. In the presence of 20% serum, coatings with a loading level of DPO0.5 or higher completely eradicated bacteria within 2 h, whereas DPO0.2 significantly reduced the bacterial load but did not achieve complete killing (Figure 3d,f). In contrast, negligible bactericidal activity was observed for all DPO‐coated surfaces in the absence of serum, consistent with the non‐bactericidal, capsule‐degrading mechanism and serum‐dependent killing of DPO71. Similar trends were observed across other substrates (Figure S11), further demonstrating that antibacterial efficiency correlates with protein loading and is dependent on serum‐mediated mechanisms.

To further investigate this dependence, we compared the antibacterial performance of DPO coatings in normal vs. heat‐inactivated serum (Figure S12). The results clearly show that DPO‐mediated bacterial killing requires active serum components. As a depolymerase, DPO71 does not directly kill bacteria but degrades bacterial CPS and EPS, thereby compromising the bacterial protective barrier. This degradation exposes the bacterial surface to host immune factors, enabling complement‐mediated killing. In contrast, heat inactivation disrupts complement activity, abolishing the bactericidal effect despite the presence of DPO.

CPS and EPS on *A. baumannii* form a protective barrier that limits complement activation by hindering C3b deposition and membrane attack complex (MAC, C5b–9) formation. By hydrolyzing CPS and EPS, DPO71 removes this barrier, facilitating complement activation and membrane permeabilization [42, 43, 44]. Our previous study further suggests that this sensitization is accompanied by disruption of oxidative phosphorylation, leading to reduced ATP production, impaired efflux pump activity, and increased reactive oxygen species (ROS) generation [45]. Together, these effects synergistically enhance membrane damage and ultimately result in efficient bacterial killing.

We next investigated the time dependence of bactericidal activity to better understand the kinetics of serum‐assisted killing. DPO2 exhibited rapid antibacterial activity, completely eliminating bacteria within 30 min at serum concentrations ≥10% and within 60 min at 5% serum. In contrast, DPO0.5 displayed substantially slower killing kinetics, achieving complete bacterial elimination only after 60 min at 20% serum. At lower serum concentrations, DPO0.5 failed to achieve complete killing at 10% serum and showed no detectable antibacterial activity at 5% serum (Figure 3e). These results indicate that both sufficient DPO71 surface density and adequate serum availability are required for rapid and complete bacterial eradication, suggesting the existence of a threshold level of capsule degradation necessary to enable efficient serum‐mediated killing.

To further evaluate the robustness of the antibacterial coating, we challenged the surfaces with a high bacterial load of up to 10^8^ CFU (Figure 3g). Despite this high bacterial load, both DPO2 and DPO4 coatings were able to eliminate nearly all bacteria, demonstrating the outstanding bactericidal ability of the DPO‐functionalized surfaces. The morphologies of bacteria on different coatings, after incubating with and without 20% serum, were visualized using SEM (Figure 3h). The top panel shows SEM images of bacteria on the DPO coating without serum, while the bottom panel illustrates the corresponding condition in the presence of 20% serum. In the presence of serum, bacteria on all DPO‐coated surfaces exhibited pronounced membrane shrinkage, collapse, and pore formation, indicating severe membrane damage. In contrast, bacteria adhering to uncoated (control) and PDA‐coated surfaces remained densely packed with intact membranes and well‐preserved capsular structures. In the absence of serum, although bacterial adhesion to DPO‐coated surfaces was observed, the number of adherent bacteria was markedly reduced, and the surrounding CPS and/or EPS layers were absent. These observations confirmed that surface‐immobilized DPO71 retains its enzymatic activity, efficiently degrading bacterial capsules. Capsule degradation alone impaired bacterial adhesion, while in the presence of serum it facilitated rapid and potent serum‐mediated bacterial killing.

Collectively, these results demonstrate that the antibacterial activity of DPO‐coated surfaces arises from a synergistic mechanism between enzymatic capsule degradation by immobilized Dpo71 and host‐derived bactericidal factors in serum. This dual action highlights the high efficacy, rapid killing kinetics, and robustness of the DPO coating, even under high bacterial challenge.

### 3.3 Biofilm Inhibition of the DPO Coatings

Biofilm formation on catheter surfaces is a major contributor to CRBSI and often leads to persistent and chronic infections in patients. We therefore investigated the ability of the DPO coatings to inhibit the formation of biofilms. As shown in Figure 3i, a thick, dense layer of bacterial cells adhered to the PDA‐coated (DPO0) surfaces, suggesting that *A. baumannii* had successfully established biofilms on the surfaces. On the other hand, substantially fewer viable bacteria were observed on the DPO‐coated surfaces, with the strongest biofilm inhibition effect evident for coatings with higher protein loading (DPO1−DPO4). Consistent antibiofilm effects were also observed when DPO coatings were applied to other material substrates in the presence of 20% serum (Figure S13), suggesting the broad applicability of this strategy. Quantitative analysis using crystal violet (CV) staining further confirmed the antibiofilm efficiency of the DPO coatings (Figure 3j,k). The DPO‐functionalized surfaces significantly slowed early biofilm formation at the 2 h time point and reduced total biofilm biomass by approximately 95% within 4 h in the presence of 20% serum. These results indicate that DPO coatings effectively impair the initial stages of bacterial adhesion and early biofilm development.

The pronounced antibiofilm activity is likely attributable to the enzymatic degradation of the CPS and EPS by surface‐immobilized DPO71, which disrupts the structural components required for stable surface attachment and biofilm maturation. In the presence of serum, capsule removal further enhances bacterial susceptibility to host immune factors, synergistically suppressing biofilm establishment.

While these observations were initially obtained using DPO‐coated 24‐well plate surfaces, we further evaluated the antibacterial performance of DPO‐coated catheters to better reflect practical conditions (Figure S14). Despite the limited contact area between the catheter surface and the bacterial suspension, the DPO‐coated catheters retained strong antibacterial activity, demonstrating their robustness in a more clinically relevant setting. Collectively, these findings demonstrate that DPO coatings provide a powerful strategy for reducing bacterial adhesion and inhibiting biofilm formation, highlighting their potential for preventing CRBSIs and other device‐associated infections.

### 3.4 Biocompatibility, Stability and Longevity of the DPO Coatings

**FIGURE 4:**
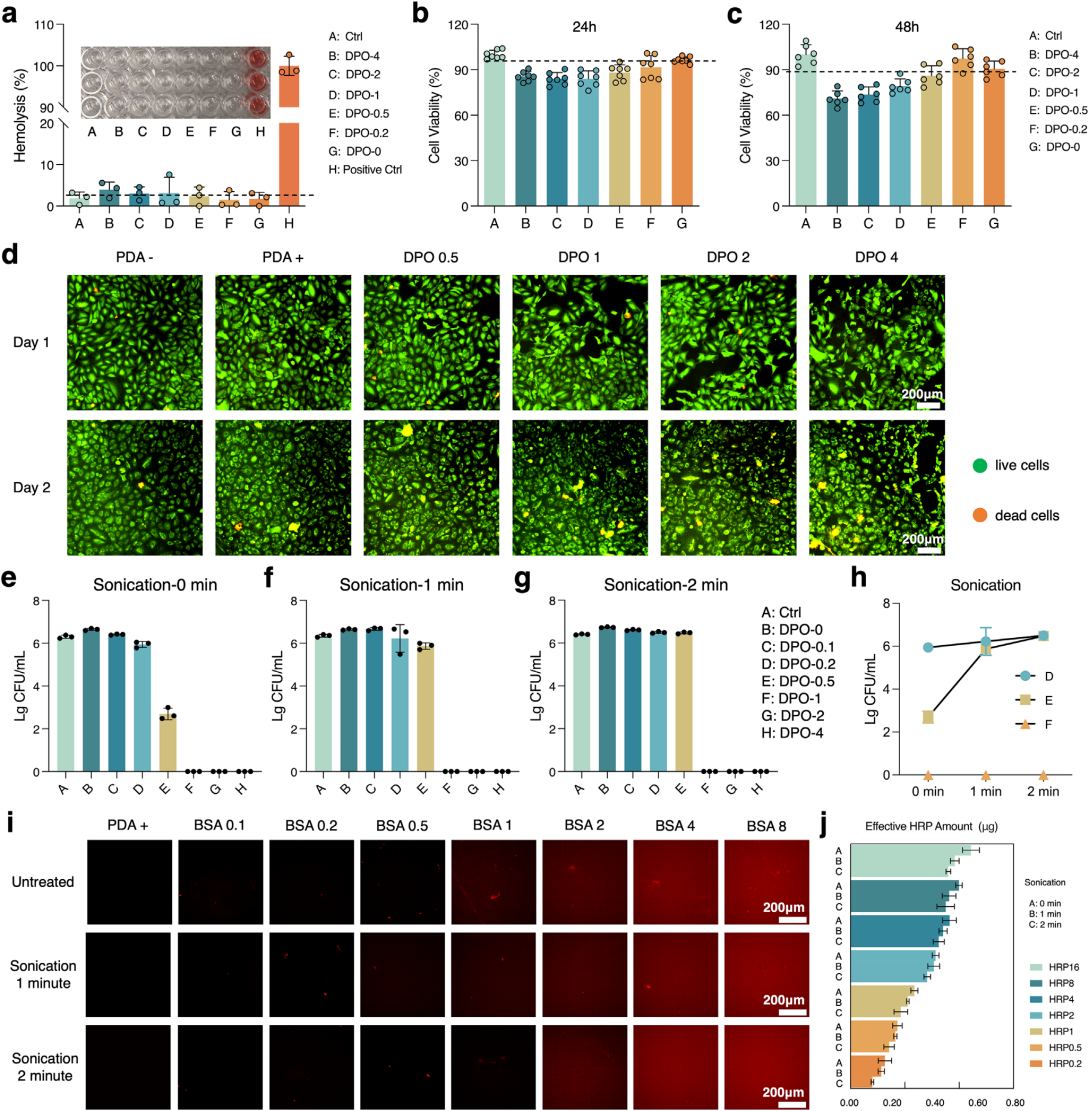
Biocompatibility and stability of the DPO coatings. (a) Hemolysis testing of the DPO coatings. (b) CCK‐8 assay of A549 cells on PDA and DPO coatings at 24 h and (c) 48 h. (d) Calcein‐AM/PI staining fluorescent images of A549 cells on PDA and DPO coatings at day 1 and day 2. (Scale bar is 200 µm.) (e–h) The antimicrobial activity of control, PDA, and DPO coatings after ultrasonication (50 kHz, 200 W) at different times (1 min and 2 min). (i) Fluorescent microscopy images of BSA conjugated with Sulfo‐Cy5 dye on the surface after ultrasonic 1 min and 2 min. (Scale bar is 200 µm.) (j) Quantification of the anti‐IgG HRP coating after ultrasonic 1 min and 2 min. Data are presented as mean ± standard deviation (s.d.).

To evaluate the promise for clinical applications, the hemolytic activity of DPO coatings was assessed. As shown in Figure 4a, all coatings exhibited low hemolysis activity (< 5%), indicating good blood compatibility. We next investigated the cytocompatibility of the DPO coating, as direct contact with human cells would be inevitable and, in some cases, desirable like in tissue engineering applications. Human lung epithelial cells (A549) were cultured on uncoated, PDA‐coated, and DPO‐coated 24‐well plates, and cell viability was determined using the CCK‐8 assay. Overall, robust cell growth was observed in all groups after 1 and 2 days of incubation. Cell viability remained above 80% after 1 day and above 70% after 2 days across all groups, demonstrating the acceptable biocompatibility of the DPO coatings (Figure 4b,c). Fluorescence microscopy was further used to examine the morphology of A549 cells on different surfaces (Figure 4d). The epithelial cells displayed normal shapes regardless of the coatings, with no significant differences in cell density among groups. By day 2, only a small number of dead cells were detected. Collectively, these results indicate that DPO coatings support cell attachment and provide a favorable environment for epithelial cell growth.

The stability of immobilized depolymerases is crucial for their practical applications. We next evaluated the stability of the DPO coatings using an ultrasonic test (50 kHz, 200 W). As shown in Figure 4e–h, the antibacterial activity of most DPO coatings remained stable with increasing sonication time, whereas a significant reduction was observed in the DPO0.5 group. This result indicates that the DPO0.5 coating represented a threshold coating density at which the antibacterial performance become sensitive to external perturbations, such as ultrasonication (Figure 4h). Nonetheless, coatings with higher DPO71 surface coverage (DPO1‐DPO4) retained potent bacterial killing capacity, achieving 100% killing even after 2 min of sonication. The retention of surface‐immobilized proteins after sonication was further examined using Sulfo‐Cy5‐labelled BSA (Figure 4i). In addition, quantification of immobilized anti‐IgG HRP coating demonstrated that the protein coatings remained largely stable under ultrasonication (Figure 4j). Although most of the depolymerase remained immobilized on the surface after sonication, the extent of DPO71 detachment increased with prolonged sonication time, suggesting that the reduced antibacterial activity observed for DPO0.5 was primarily due to partial enzyme removal from the surface. Overall, despite a modest decrease in surface‐bound DPO71 under harsh mechanical stress, the PDA‐based DPO coating strategy exhibits good stability and robustness, supporting its potential as a simple, effective, and broadly applicable platform for creating functional macromolecule‐bonded surfaces.

In addition, we evaluated the longevity of the DPO coating (Figure S15). According to the results, the DPO solution exhibited excellent storage stability under refrigeration, with no significant loss of activity after 4 weeks at 4°C (Figure S15a). Similarly, the DPO coating (stored in PBS) maintained its antibacterial activity after 2 weeks of storage at 4°C (Figure S15b). These findings indicate that storing at low temperature and hydrated conditions is effective in preserving the functionality of the DPO coating. The durability of the DPO coating was also confirmed through repeated antibacterial assays, with no reduction in activity observed after six cycles (Figure S15c), demonstrating its reusability and sustained performance.

In contrast, environmental stress conditions had a detrimental effect on the DPO coating. Drying it at 37°C for 4 h or subjecting it to moist heat sterilization at 115°C for 30 min resulted in a complete loss of bactericidal activity (Figure S15d,e), indicating that dehydration and elevated temperatures irreversibly inactivate the protein. Taken together, these findings demonstrate that the activity of DPO coatings is highly dependent on storage and handling conditions. While refrigerated and hydrated environments enable the coating to retain its antibacterial activity and reusability for up to approximately one month, drying and heat exposure lead to irreversible loss of function.

### 3.5 Practicality of DPO Coatings Under Simulated Application Conditions

**FIGURE 5:**
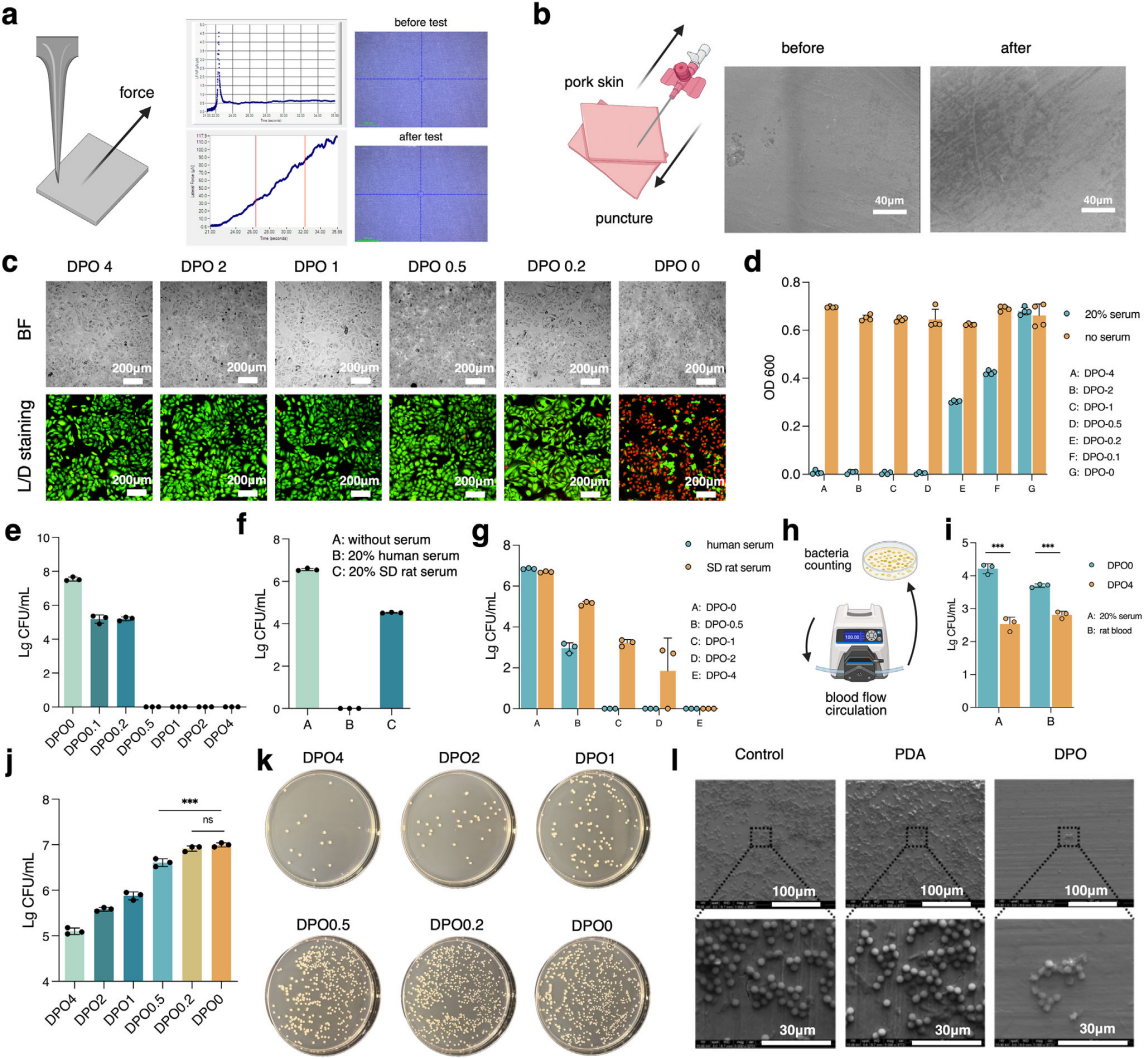
Ex Vivo application of DPO coating on different surfaces. (a) Schematic diagram (Created with Biorender.com) and results of nano‐scratch testing on the surface of BSA@PDA@PU. (b) Schematic diagram (Created with Biorender.com) and SEM imaging of indwelling needle catheters with DPO coating. (Scale bar is 40 µm.) (c) Calcein‐AM/PI staining fluorescent images of A549 cells in the cell‐bacteria co‐culture system on DPO coating. (Scale bar is 200 µm.) (d) OD 600 value of the supernatant after 24 h co‐culture. (e) Bacterial count from the surface in different groups after 12 h co‐culture. (f) Antibacterial activity of DPO solution in the presence of rat serum. (g) Antibacterial activity of DPO coating in the presence of rat serum. (h) Schematic diagram of ex vivo blood circulation system (Created with Biorender.com). (i) Antibacterial activity of DPO coating on the catheter in the circulation system in vitro. (j) Antibacterial activity of DPO coating in the rat whole blood‐bacteria co‐culture system and (k) photos of colonies from different groups. (l) SEM images of control, PDA, and DPO (DPO‐C) coatings after incubating *A. baumannii* with blood overnight. (Scale bar = 100 µm in the first row and 30 µm in the second row) Data are presented as mean ± s.d. (*n* = 3 independent samples) and analyzed with Student's t‐test (Figure 5i) and one‐way ANOVA followed by Tukey's multiple comparisons test (Figure 5j). ^*^p < 0.05, ^**^p < 0.01, ^***^p < 0.001, and ^****^p < 0.0001.

To assess the mechanical robustness of the DPO coating under conditions relevant to catheter use, we first performed nano‐scratch testing to simulate the shear stress experienced during catheter insertion. The lateral force increased nearly linearly throughout the scratching process, indicating that the coating remained well adhered to the substrate without delamination (Figure 5a). Optical images of the scratched regions further confirmed the integrity of the coating. Building on this controlled mechanical evaluation, we next examined coating durability under more realistic puncture conditions. Repeated puncture testing of DPO‐coated indwelling needle catheters through pork skin demonstrated that the coating was well retained, as evidenced by SEM imaging, even after multiple puncture events (Figure 5b). Together, these results demonstrate that the DPO coating is mechanically robust under both simulated shear stress and practical puncture conditions, supporting its suitability for real‐world catheter applications.

Next, we investigated the selectivity of the DPO coatings toward epithelial cells and bacteria using a co‐culture system. Bacterial culture was first incubated on DPO‐coated wells for 2 h, followed by the addition of A549 cells in culture medium. After overnight co‐incubation, surface‐associated bacteria were quantified, and A549 cell viability was visualized using a Calcein‐AM/PI assay. The fluorescent images revealed that DPO coatings effectively killed bacteria while simultaneously supporting healthy epithelial cell growth (Figure 5c). The OD_600_ values of the supernatants increased markedly in the DPO0 to DPO0.2 groups, indicating sustained bacterial growth under these conditions, whereas OD_600_ values for DPO0.5–DPO4 remained low (<0.1), reflecting effective suppression of bacterial proliferation (Figure 5d). Consistently, while no viable bacteria were detected on surfaces with higher DPO coating coverage (DPO0.5‐DPO4), significant bacterial colonization was observed in PDA(DPO0), DPO0.1, and DPO0.2 groups (Figure 5e). This pattern indicates that insufficient DPO coverage fails to achieve complete bacterial eradication, thereby allowing continued bacterial growth both in suspension and on the material surface. Nonetheless, these results demonstrate that DPO coatings at sufficient coverage exhibit strong antibacterial selectivity while preserving epithelial cell viability, highlighting their suitability for applications requiring concurrent bacterial suppression and host‐cell compatibility.

Next, blood infection models were employed to evaluate the translational potential of the DPO coatings for clinical applications in mitigating CRBSIs. We first assessed the antibacterial activity of DPO71 solution and DPO‐coated surfaces in the presence of rat serum. Although rat serum exhibited lower antibacterial efficiency than human serum, the DPO4 coating completely killed all bacteria within 2 h in 20% rat serum, demonstrating the cross‐species universality of the DPO coating strategy (Figure 5f,g). Antibacterial performance of the DPO coating was further evaluated in a rat blood–bacteria co‐culture system with constant shaking (200 rpm). Compared with the PDA control (DPO0), the DPO4 coating eliminated approximately 95% of bacteria (Figure 5j,k). Surface colonization further supported these results (Figure 5l), as extensive bacterial coverage was observed on uncoated and PDA‐coated surfaces, whereas only sparse bacterial cells with disrupted morphology were detected on the DPO‐coated surface. Furthermore, SEM images showed no observable adhesion of blood cells or other cellular components on the DPO‐coated surface. The absence of cell adhesion suggests that the coating does not promote surface‐induced coagulation or thrombus formation under the tested conditions. Quantitative bacterial counts recovered from both the blood and surface‐associated fractions consistently confirmed the potent antibacterial activity of the DPO coating under blood‐contact conditions (Figure S16). Beyond static blood contact with shaking, a dynamic blood circulation system was established to closely mimic physiological conditions. Under continuous circulation for 24 h, DPO‐coated catheters achieved approximately 98% bacterial killing in 20% human serum and 90% killing in rat blood, further demonstrating robust antibacterial performance under flow conditions (Figure 5h,i).

Importantly, in both static and dynamic blood‐contact experiments, no adhesion or accumulation of blood cells was observed on DPO‐coated surfaces, indicating that the DPO coating does not induce blood clot formation and is compatible with blood‐contacting applications.

### 3.6 Bacterial Infection and Treatment Models in the *Galleria Mellonella* Larvae

**FIGURE 6:**
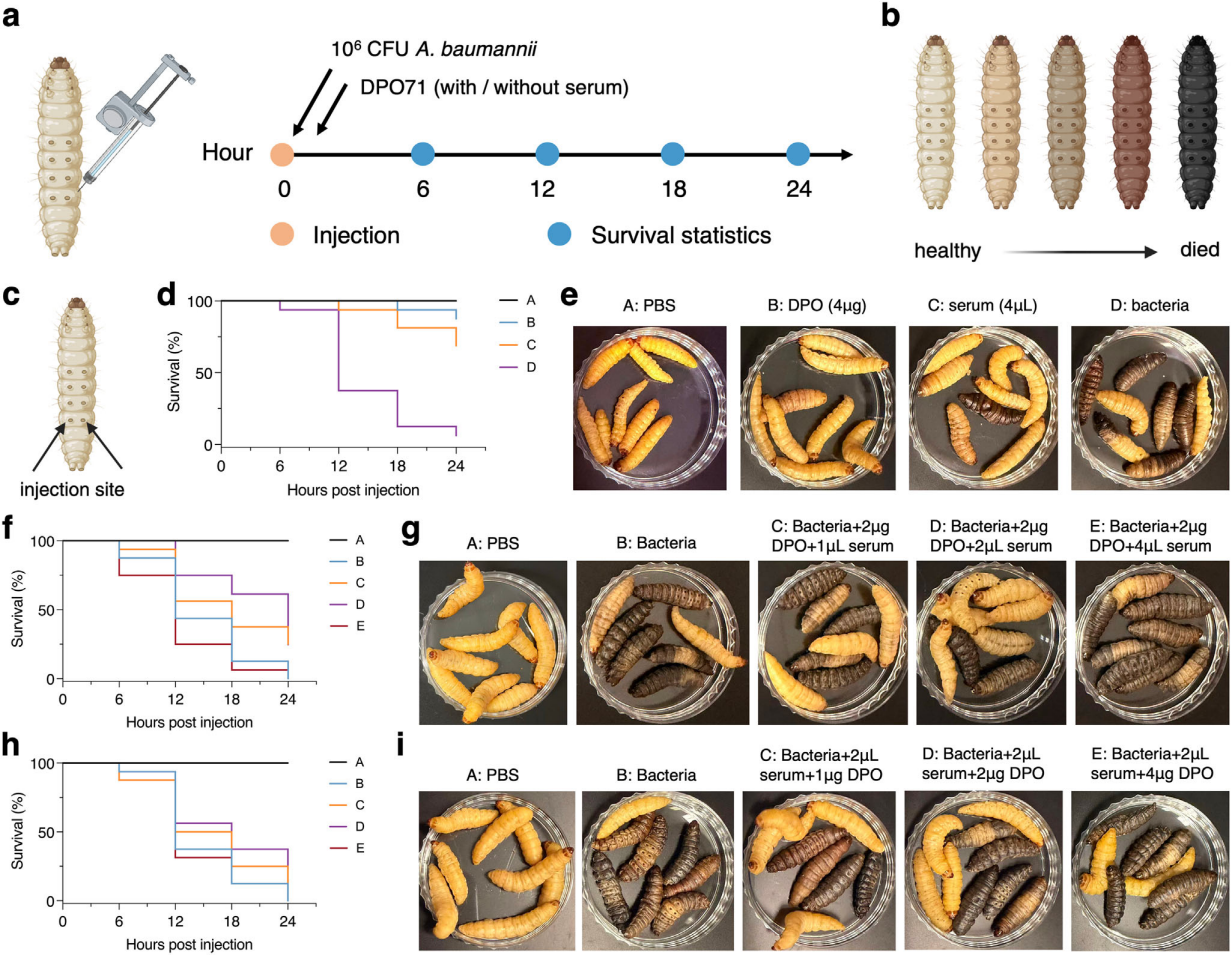
Bacterial infection and treatment models in the *Galleria mellonella* larvae. (a) A diagram illustrating *Galleria mellonella* bacterial infection and treatment. Created with Biorender.com. (b) A diagram illustrating the health status of *Galleria mellonella*. Created with Biorender.com. (c) Schematic diagram of the injection site. Created with Biorender.com. (d) Survival statistics of *Galleria mellonella* larvae after different treatments and (e) photographs at 12 h. (f) Survival statistics of *Galleria mellonella* larvae after different treatments when the serum concentration is different and (g) photographs at 12 h. (h) Survival statistics of *Galleria mellonella* larvae after different treatments when the DPO concentration is different and (i) photographs at 12 h. (*n* = 16 biological independent samples in each group).

*Galleria mellonella* larvae are widely used as a convenient in vivo model for assessing drug toxicity and bacterial virulence, and for the preliminary evaluation of antimicrobial efficacy. Figure 6a–c illustrates the experimental design used in this study, including infection and treatment workflows, health‐status scoring, and injection sites. We first assessed the safety of DPO71 and serum in *G. mellonella*. All larvae were injected with 20 µL of solutions containing different components. As shown in Figure 6d,e, DPO71 exhibited good in vivo safety, with high survival comparable to the PBS control. In contrast, a small proportion of larvae displayed intolerance when exposed to 20% serum, indicating a limited systemic tolerance to high serum concentrations in this model.

Next, we conducted a preliminary assessment of the in vivo antibacterial efficacy of DPO71 using the *Galleria mellonella* infection model (injected with 1 × 10^6^ CFU bacteria). At a DPO71 dose of 2 µg, co‐injection with 2 µL of human serum significantly delayed larval mortality. However, a higher serum level (4 µL) not only failed to confer therapeutic benefit but led to more rapid death compared with the untreated infected larvae (Figure 6f,g). This outcome likely reflected a combined toxic effect resulting from bacterial virulence factors and serum components, which accelerated larval death. Interestingly, as shown in Figure 6h,i, high concentrations of DPO71 in the presence of 2 µL serum also did not yield improved therapeutic outcomes. Larval survival initially increased as the DPO71 dose rose to 2 µg, but a higher dose (4 µg) resulted in accelerated mortality. These findings suggest that excessively rapid bacterial killing may trigger the extensive release of bacterial components, thereby exacerbating systemic toxicity and hastening death in *G. mellonella*.

Overall, though *G. mellonella* is not an ideal in vivo model for fully recapitulating therapeutic outcomes of antibacterial treatment in higher organisms, it effectively demonstrates the in vivo safety of DPO71 and supports the feasibility of DPO‐based antibacterial strategies for in vivo applications.

### 3.7 Subcutaneous Catheter Implantation Models in Mice

**FIGURE 7:**
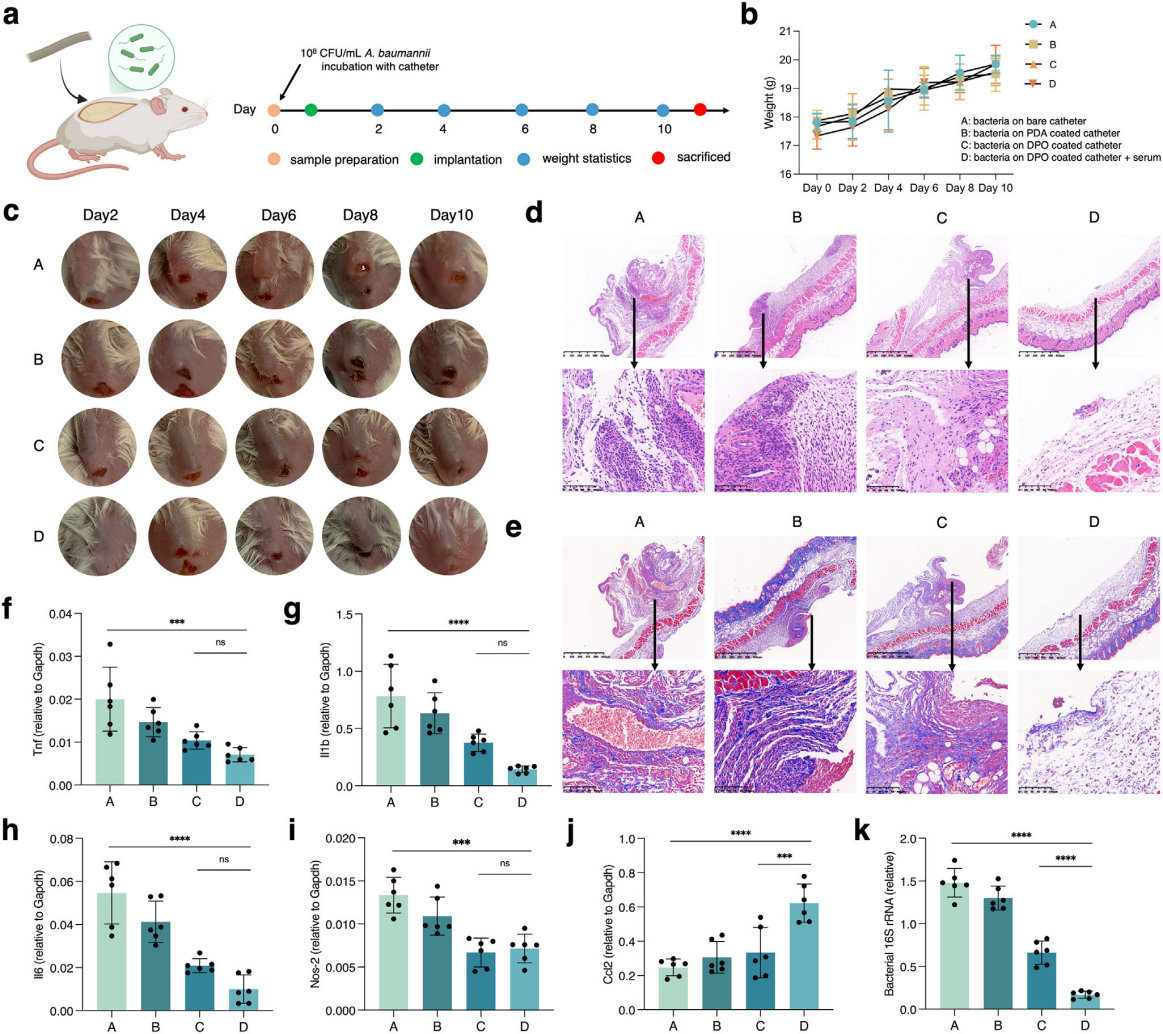
Subcutaneous catheter implantation models in mice. (a) Schematic diagram of experimental design. Created with Biorender.com. (b) Body weight of mice in different groups at different time points. (c) Images of catheter implantation sites at different times in each group. (d) H&E staining and (e) Masson staining of skin tissue at the catheter implantation site. (Scale bar = 625 µm in the first row and 100 µm in the second row) (f–i) qRT‐PCR analysis of Tnf (TNF‐α), Il1b (IL‐1β), Il6 (IL‐6), Nos‐2, Ccl2, and bacterial 16S rRNA ‌expression in the skin tissues at the catheter implantation site. Data are presented as mean ± s.d. (*n* = 6 independent samples) and analyzed with one‐way ANOVA followed by Tukey's multiple comparisons test. ^*^ *p* < 0.05, ^**^ *p* < 0.01, ^***^ *p* < 0.001 and ^****^ *p* < 0.0001.

Subcutaneous implantation of bacteria‐loaded catheters in mice provides a more clinically relevant evaluation model, as the involvement of host immunity and body fluid (similar to serum components) allows a more comprehensive assessment of the therapeutic potential of DPO coatings. Figure 7a illustrates the experimental design of the catheter implantation mouse model. Briefly, catheter fragments (uncoated, PDA‐coated, and DPO‐coated) were first immersed in bacterial suspension containing 1 × 10^7^ CFU bacteria for 2 h to allow bacterial adsorption. Then, the contaminated catheter fragments were implanted subcutaneously into the tissue of the dorsal tissue of mice.

Throughout the 10‐day observation period, all mice exhibited stable body‐weight gain, demonstrating that the subcutaneous implantation did not induce systemic toxicity and infection (Figure 7b). Macroscopic examination of the implantation sites revealed the presence of secondary wounds in mice implanted with uncoated and PDA‐coated catheter fragments at early time points, but these lesions did not progressively worsen over time. To further distinguish the inflammatory response caused by catheter implantation from that induced by bacterial infection, an additional set of animal experiments was conducted (Figure S17). The results showed that catheter implantation alone elicited a measurable inflammatory response compared to normal tissue. However, this response was significantly lower than that associated with bacterial infection. These findings indicated that the level of inflammation attributed to the catheter itself is relatively limited and does not dominate the overall inflammatory outcome in infected conditions.

At the end of the experiment, mice were euthanized, and skin tissues surrounding the implantation sites were collected for further examination. H&E staining revealed distinct differences among different treatment groups (Figure 7d). Large amounts of infiltrated lymphocytes were observed in mice implanted with uncoated (Group A) and PDA‐coated (Group B) catheter fragments, especially in the dermis, while immune cell infiltration was markedly reduced in mice implanted with DPO‐coated catheter (Group C) and became sparser in mice co‐administered with human serum (Group D). Consistently, Masson's staining demonstrated pronounced collagen deposition and dermal fibrosis in Groups A–C, accompanied by persistent lymphocyte infiltration, while Group D exhibited significantly attenuated fibrotic responses and milder inflammatory features (Figure 7e).

The inflammatory responses were further elucidated at the molecular level by performing RT‐qPCR to quantify inflammation‐ and immune‐associated genes in tissues surrounding the implantation sites. The DPO coating significantly reduced the mRNA expression levels of the pro‐inflammatory cytokines, including Tnf (TNF‐α), Il1b (IL‐1β), and Il6 (IL‐6), with a more pronounced suppressive effect observed in the presence of serum (Figure 7f–h). In addition, expression of Nos‐2, a marker of inflammatory immune activation, was markedly reduced, indicating effective suppression of excessive inflammatory responses that are known to impede tissue repair (Figure 7i). Concurrently, Ccl2 expression was significantly elevated in mice with DPO‐coated implants in the presence of serum (Figure 7j), suggesting enhanced chemokine‐mediated recruitment of monocytes/macrophages that support bacterial clearance and contribute to coordinated immune surveillance and early tissue repair, rather than uncontrolled inflammation.

Quantitative analysis of bacterial 16S rRNA further revealed that the bacterial burden in Group D was reduced to only ∼10% of that observed in Group A (uncoated). Collectively, these results demonstrated the in vivo applicability of the DPO coating and indicated that its primary function is to rapidly kill bacteria while modulating the local immune microenvironment via attenuating excessive inflammatory signals and promoting appropriate immune cell recruitment to facilitate bacterial clearance. This balanced immune response prevents the persistence of chronic inflammation, dermal fibrosis, and prolonged immune cell infiltration typically associated with implant‐related bacterial infection.

### 3.8 Study Limitations and Future Perspectives

As summarized in Table S2, the DPO‐based coating differs fundamentally from conventional antibacterial coating strategies, such as leaching‐based systems, contact‐killing surfaces, and intact phage coatings. Rather than directly killing bacteria, this approach degrades polysaccharide barriers to promote bacterial susceptibility to host immune factors and antibiotics. While this mechanism reduces selective pressure for antimicrobial resistance, several limitations of the current study should be acknowledged.

First, the in vivo model used does not fully capture the pathophysiological features of clinically relevant CRBSIs. Although an ex vivo blood circulation model was incorporated to more closely mimic blood contact conditions and successfully validated the antimicrobial effectiveness of the DPO coating under dynamic flow, this system cannot fully reproduce the complex host responses present in living organisms, such as vascular immune signaling, systemic inflammation, and long‐term biofilm‐host interactions. Nevertheless, by combining in vivo implantation studies with a physiologically relevant ex vivo circulation platform, the present work provides an important translational bridge that rigorously establishes both the antibacterial efficacy and immunomodulatory function of the DPO coating across complementary biological settings. Building on this strong mechanistic and preclinical foundation, future validation in advanced in vivo CRBSI animal models, such as intravascular catheter implantation with sustained blood flow and bloodstream bacterial challenge, will further clarify the durability, safety, and clinical applicability of the DPO coatings.

In addition, DPO71 exhibits species‐specific activity against *A. baumannii*, which limits its effectiveness in polymicrobial catheter‐associated infections. To broaden the antibacterial spectrum, future strategies may focus on developing combination approaches. These could include incorporating multiple depolymerases targeting different bacterial species or capsule types, or integrating DPO‐based coatings with complementary antibacterial modalities, such as antibiotic‐releasing or contact‐killing coatings, to achieve broader antibacterial coverage.

Despite these limitations, the present study establishes a strong mechanistic and preclinical foundation for depolymerase‐based coatings. By integrating in vitro, ex vivo, and in vivo models, this work provides a valuable translational framework supporting the development of DPO‐functionalized surfaces for preventing device‐associated infections.

## 4 Conclusions

Increasing attention has been drawn to the development of phage‐encoded depolymerases as antivirulence agents for the management of sepsis because their bacterial killing capacity rely on host immune components, such as serum factors or neutrophil‐mediated killing. In this work, we present the first demonstration of immobilizing this class of antibacterial enzymes onto catheter surfaces via a simple yet robust mussel‐inspired PDA chemistry to mitigate CRBSI. Following surface immobilization on the surface, the depolymerase DPO71 retained its bioactivity and exhibited excellent antimicrobial and antibiofilm activities in 20% serum. In addition, the DPO coating demonstrated good mechanical stability under ultrasonication and higher shearing upon implant insertion, high selectivity toward bacterial killing, and excellent biocompatibility. A series of simulated application scenarios further confirmed the practical feasibility and cross‐species universality of the DPO coating strategy. Moreover, both *Galleria mellonella* infection model and the mouse subcutaneous catheter implantation model demonstrated the in vivo antibacterial efficacy of DPO coatings, providing compelling evidence for their further clinical development. These encouraging findings establish PDA‐mediated immobilization of phage depolymerases as a promising and versatile surface‐engineering strategy, offering an effective alternative for preventing CRBSI and other bacterial infections associated with indwelling medical devices.

## Author Contributions

**Lingkai Dong**: Conceptualization, Methodology, Formal analysis, Investigation, Visualization, Writing – original draft. **Xingjin Li**: Methodology, Investigation. **Tao Hu**: Methodology. **Qianqian Lu**: Methodology. **Xiaoxu Zhang**: Methodology. **Xi Chen**: Methodology, Investigation. **Yifan Luo**: Investigation. **Zhong Zuo**: Conceptualization, Writing – review & editing. **Ronald Man Yeung Wong**: Conceptualization, Writing – review & editing. **Miao Xu**: Conceptualization, Methodology, Formal analysis, Investigation, Writing – original draft. **Tiancong Zhao**: Conceptualization, Resources, Writing – review & editing.  **Sharon Shui Yee Leung**: Conceptualization, Resources, Supervision, Project administration, Funding acquisition, Writing – review & editing.

## Conflicts of Interest

The authors declare no conflicts of interest.

## Supporting information




**Supporting File**: advs77319‐sup‐0001‐SuppMat.docx.

## Data Availability

The data that support the findings of this study are available from the corresponding author upon reasonable request.
