# Supplementary material for: One‐Step Immobilized Phage Depolymerase Coatings for Catheter‐Related Bloodstream Infections
Source: Adv Sci (Weinh). 2026 Aug 21:e77319. Online ahead of print. doi: 10.1002/advs.77319 (PMC13495948; doi:10.1002/advs.77319)
Supplement: Supplementary file 1 [file ADVS-9999-e77319-s001.docx]

**Supporting Information** for

**One-step Immobilized Phage Depolymerase Coatings for Catheter-Related Bloodstream Infections**

Lingkai Dong ^a^, Xingjin Li ^b^, Tao Hu ^c^, Qianqian Lu ^b^, Xiaoxu Zhang ^a^, Xi Chen ^d^, Yifan Luo ^a^, Zhong Zuo ^a^, Ronald Man Yeung Wong ^e^, Miao Xu ^a^*, Tiancong Zhao ^b^*, Sharon Shui Yee Leung ^a^*

^a^ School of Pharmacy, The Chinese University of Hong Kong, Shatin, Hong Kong, China

^b^ College of Smart Materials and Future Energy, Department of Chemistry, Fudan University,

Shanghai, China

^c^ Institutes of Biomedical Sciences, Shanghai Key Laboratory of Medical Epigenetics, Fudan University, Shanghai, China

^d^ Department of Chemistry, The Chinese University of Hong Kong, Shatin, Hong Kong, China

^e^ Department of Orthopaedics & Traumatology, The Chinese University of Hong Kong, Hong Kong, China

**Figure S1.** The photos of PDA coating on different materials (PU, glass and titanium).

**Figure S2.** The photos of PDA and DPO71 coating on different materials (titanium, PDMS and glass).

**Figure S3.** The XPS results of Ti 2p from titanium plate, PDA@Ti and BSA@PDA@Ti.

**Figure S4.** Survey of the XPS signals from polyurethane plate, PDA@PU and BSA@PDA@PU.

BSA@PDA@Ti

PDA@Ti

**Figure S5.** High-resolution XPS spectra of C 1s from PDA@Ti and BSA@PDA@Ti.

BSA@PDA@PU

PDA@PU

**Figure S6.** High-resolution XPS spectra of N 1s from PDA@PU and BSA@PDA@PU.

**Figure S7.** SEM imaging of the surface morphology from glass (SiO_2_), PDA@SiO_2_ and BSA@PDA@SiO_2_. (Scale bar = 40 μm in the first row and 10 μm in the second row)

**Figure S8.** Fluorescent imaging of the Cy5-BSA on the surface of BSA@PDA@SiO_2_. (Scale bar = 200 μm)

**Figure S9. Characterization of the DPO coatings.** (**a**) Raman scattering results of glass (SiO_2_), PDA@SiO_2_ and DPO@PDA@SiO_2_. (**b**) Survey of the XPS from PDA@SiO_2_ and DPO@PDA@SiO_2_. (**c**) The XPS results of S 2p from PDA@SiO_2_ and DPO@PDA@SiO_2_. (**d**) High-resolution XPS spectra of C 1s from PDA@SiO_2_ and DPO@PDA@SiO_2_. (**e**) SEM imaging of the surface morphology from SiO_2_, PDA@SiO_2_ and DPO@PDA@SiO_2_,(Scale bar = 1 μm) and (**f**) EDS mapping of the DPO@PDA@SiO_2_ surface. (Scale bar = 50 μm)

**Figure S10:** protein quantification via the measurement of the fluorescence in the solution.

**Figure S11.** Photos of colonies from different samples treated with no serum and serum groups.

**Figure S12.** The role of the complement system in serum-mediated bactericidal activity of DPO.

**Figure S13.** Anti-biofilm activity of the uncoated surface (control), and the PDA and DPO coatings with 20% serum under confocal microscopy. (Scale bar = 200 μm).

**Figure S14:** The antibacterial efficacy of DPO-coated catheter *in vitro*.

**Figure S15: Longevity and stability of the DPO coating. (a)** Bactericidal activity of the DPO solution stored at 4°C and evaluated on Day 0 and Day 28. **(b)** Bactericidal activity of DPO coatings after storage at 4°C for two weeks. **(c)** Repeated evaluation of the bactericidal activity of the DPO coating over six cycles at 12-hour intervals. Bactericidal activity of the DPO coating after **(d)** 4 hours of drying at 37°C and **(e)** sterilization at 115°C for 30 min.

Ctrl	PDA	DPOC

**Figure S16.** Bacterial counts from the medium and the surface in different groups.

**Figure S17: Evaluation of basal inflammation induced by catheter implantation.** (**a-d**) qRT-PCR analysis of Tnf (TNF-α), Il1b (IL-1β), Il6 (IL-6) and Nos-2 in the skin tissues at the catheter implantation site. Data are presented as mean ± s.d. (n = 6 independent samples) and analyzed with one-way ANOVA followed by Tukey's multiple comparisons test.

**Table S1.** qPCR primers and sequences used in the study.

| **Name** | **Sequence (5’ to 3’)** |
| --- | --- |
| Tnf-F  Tnf-R  Il1b-F  Il1b-R  Il6-F  Il6-R  Nos2-F  Nos2-R  Arg1-F  Arg1-R  Ccl2-F  Ccl2-R  Tgfb1-F  Tgfb1-R  Bacterial 16S rRNA-F  Bacterial 16S rRNA-R | CCCTCACACTCAGATCATCTTCT  GCTACGACGTGGGCTACAG  GCAACTGTTCCTGAACTCAACT  ATCTTTTGGGGTCCGTCAACT  TAGTCCTTCCTACCCCAATTTCC  TTGGTCCTTAGCCACTCCTTC  GTTCTCAGCCCAACAATACAAGA  GTGGACGGGTCGATGTCAC  CTCCAAGCCAAAGTCCTTAGAG  AGGAGCTGTCATTAGGGACATC  TTAAAAACCTGGATCGGAACCAA  GCATTAGCTTCAGATTTACGGGT  CTCCCGTGGCTTCTAGTGC  GCCTTAGTTTGGACAGGATCTG  ACTCCTACGGGAGGCAGCAGT  TATTACCGCGGCTGCTGGC |

**Table S2**. **Comparison of representative antimicrobial catheter coating technologies with the DPO coating in this study**
